# PSYCHO-EMOTIONAL CARE IN A NEONATAL UNIT DURING THE COVID-19 PANDEMIC

**DOI:** 10.1590/1984-0462/2020/38/2020119

**Published:** 2020-05-29

**Authors:** Denise Streit Morsch, Zaira Aparecida de Oliveira Custódio, Zeni Carvalho Lamy

**Affiliations:** a National Consultant of the Kangaroo Method, Ministry of Health, Brasília DF, Brazil.; b Hospital Universitário, Universidade Federal de Santa Catarina, Florianópolis, SC, Brazil.; c Public Health Department, Universidade Federal do Maranhão - São Luís, MA, Brazil.

Arduous times. Although the Covid-19 pandemic caused by SARS-CoV-2 has, until now, affected relatively few newborns (NB),^1^ it has induced intense and disorganizing changes for neonatal care, influencing the bonding and neurosensory protection practices so hard-won over the past years.^2^

With the presence of SARS-CoV-2, the current context requires distancing and reduced movement of people. Namely, it demands the reformulation of procedures and practices, leading to the need for new strategies to ensure care. Published documents suspended the visits from grandparents, siblings, and other individuals who comprised the support network, guaranteeing the exclusive access to the asymptomatic mother and/or father, after daily and safe checks at the entry to the Neonatal Intensive Care Unit (NICU).^3^ ^,^ ^4^

The challenge for neonatal teams is ensuring the safety of the NB, the NB's parents, and their own, without, however, departing from the basic principles of humanized care, which have guided the neonatal care in Brazil.^2^ It is essential to understand and fulfill the requirements imposed by this moment in the world to ensure the needed adaptations, aiming at protecting the trinomial NB, family, and health team.

To this end, it is important to consider care, regarded herein as intensive, focusing on supporting the NB, the NB's parents, and the health team. The experiences of countries that first faced this disease have shown this concern. Wang et al. reported that, in neonatal units, the stress of parents and the staff is high, and that social workers and psychologists must assist both.^5^

## INTERVENTION STRATEGIES TARGETED AT NEWBORNS FOR ROUTINE CARE IN NEONATAL UNITS

In times of social distancing, the parents' unrestricted access to and presence in the neonatal unit are hindered. Many situations might lead to the parents' absence; for instance, mothers and/or fathers who are symptomatic, tested positive, or had contact with someone infected with SARS-CoV-2, those with other children and/or family members in the risk group, who live far or have transport difficulties, among others.^3^ According to Canvasser (*apud* Furlow), the effects of separating mothers and babies have been devastating.^6^ Moments of crisis require deliberated, flexible, and unique attitudes. These changes involve the work of the entire team in a process of cooperation and mutual support.

Keeping the same professionals in the care of each NB, on each shift, gives the patient a reference to recognize the routine care, the voice and touch of each professional, acting as a source of safety and trust in the face of so many changes. Mathelin^7^ emphasizes the importance of the way of touching the NB, responding to their gaze, and "addressing them, as a human addressing another human, filled with feelings, thoughts, and desires." Being fully with the NB in such a delicate moment of their lives is a compassionate attitude, as well as a neonatal health intervention that promotes their development. The offer of holding and handling each baby is a source of not only biological but also emotional support. The inner availability of the professional emerges upon the affection, the gaze, the gestures, and all aspects involved in the care relation.

Besides this bodily contact with the NB, the professional uses verbal contact by describing what is happening. The words give the NB a meaning to what they are living. Introducing ourselves, explaining the care that will be carried out, and telling the NB where they are and why their parents are not with them allow them to recognize their personal history.^8^ By using the spoken word, we give the NB an understanding of what they are experiencing in a situation of sickness and separation from their mother, father, and family.^9^

Maternal death is another troublesome situation for babies. Cyrulnik^10^ explains that

an early loss in the life of little ones leads to catastrophe when there is no emotional substitute. When an attachment figure disappears, a huge part of their sensory world vanishes. The baby's biological surroundings permanently lose their auditory, tactile, olfactory, and visual stimuli because the other is no longer present.

Words must come into action once again, bringing, along with the narrative, the presence of another family caregiver. The intent is not leaving the baby alone, without the surroundings marked by family culture.

The number of professionals who provide care for the NB should be the lowest possible. Support to the family and especially the interaction between the team and the family member who will take over the NB's care should be guaranteed. Staying beside the NB, looking at them, speaking softly, and supporting their body provide the necessary integration experience in moments of extreme vulnerability, and, according to the pediatrician and psychoanalyst Winnicott,^11^ ensure the NB's well-being.

## INTERVENTION STRATEGIES TARGETED AT FAMILIES FOR ROUTINE CARE IN NEONATAL UNITS

The entire team is responsible for facilitating the presence of the mother and/or father, whenever possible, and supporting them so they can also offer this outline of senses and meanings to the baby, stimulating the NB emotionally and using words to situate what they are experiencing.

In the parents' absence, the team must ensure communication. Cell phone use in the neonatal unit, which has always been restricted, can be an important tool in this moment of crisis, shortening the distance between the family and the baby. However, its entry in the neonatal unit must be done with caution, rigorously complying with the rules of each location. Wrapping the device in plastic film after cleaning it has been recommended.

A routine of contact mediated by the team might be established through recorded or read messages, which can also be proposed to siblings and other family members, such as grandparents. Photos and/or video records, short descriptions about how the baby is behaving, their traits, and routine can be sent to the parents.

When present, parents may be encouraged to capture images with their cell phones to share with the family, and the team must instruct them on how to clean the device and that their records should not involve other babies, families, or the team.

Special interventions should be planned in the case of death in this context, which makes the situation even harder and sadder. Everything that has been built to facilitate the connection and the mourning process will have to be adapted at this moment, when the pain of separation between the family and the baby is unimaginably high. How to experience the separation caused by death when the closeness was limited or virtually non-existent? How to deal with this pain when it is not possible to see, touch, dress, hold a vigil for the baby? This has become the reality of parents who lost their NB children to this pandemic.

Mourning rituals are suspended, and supportive physical contact is not recommended. Wakes have fixed, minimal, and insufficient time. The impossibility of ritualizing the child's death will leave deep scars in parents and relatives. Cyrulnik^10^ highlights: "When the losses are neither accepted nor signified, the mourner can only withdraw to lessen their suffering. In this case, the loss is not mourning but a hole in the soul, an emptiness without representation." This situation leads to great psychic distress and can be traumatic, resulting in depressive disorders, anxiety, post-traumatic stress, and complicated grief, as described by Muza et al.^12^

It will be up to the neonatal unit professionals to assist the parents and relatives who lost their babies without even knowing them. We suggest some actions that could be performed, considering the uniqueness of each mother and father, their family values, as well as the desire to carry them out: providing photos and videos of the NB alive; reading or playing messages from the family to the NB; instructing the parents to explain the conditions of the NB to other family members; after death, asking the parents to bring clothes and/or a small toy to accompany the baby.

If possible, the family members who live in the same household can gather and perform a mourning ritual for the NB. It might be important to advise them not to rush to dispose of clothes and furniture they prepared for their child, allowing themselves time to prepare for this moment.

The team can also deliver identification cards that belonged to the baby and a report written by the professionals who looked after the NB during their stay in the neonatal unit, with accounts of special situations. It is also essential to give family members the opportunity of returning to talk with the team, if they so wish, a few days after death, offering information and support.

## CARE STRATEGIES FOR THE CAREGIVER

The neonatal unit staff is facing a huge emotional challenge. The strategies discussed in this editorial increase this challenge. However, empathizing with other humans, enabling them to experience an expression of love in such a painful moment of their lives, can generate a feeling of fulfillment and peace with what could be provided, given the real restrictions imposed.

It is crucial to give voice to the health team and their requests. Used to well-defined protocols, the professionals face something still unknown. Comings and goings between different hospitals and the risk of infection or of transmitting the disease to family members lead to grueling routines of care, hygiene, and equipment donning and doffing, often exceeding their shifts and the time for them to go back home.

Team discussion and the support of the psychology and social service departments are important for the staff to be confident in their decisions regarding recent rules. Dialogues with other professionals who understand and validate their intentions are essential.

All these issues are present in the expressions and the frequent pleadings of health professionals in social networks: "We stay here for you, please stay at home for us." We share these concerns, fears, and fatigue. We believe that the support between professionals makes a difference. We can feel it when a hand reaches out to help handle a tube, place catheters, in the head that rests on another's shoulder to have a better view of the baby.

Also, pay attention to the NB's body responding to your handling. Look out for expressions of comfort when you treat them. Neither you nor these babies will ever forget these experiences; they will be fleeting but intense memories, offering a shared holding.

Moreover, take care of your own body. Accept suggestions about exercises that can be performed in the neonatal unit environment. Minutes of silent or guided meditation that trigger breathing awareness and mindfulness, as well as the repetition of mantras or prayers connected to your beliefs, are practices that have proven to be beneficial in promoting relaxation, concentration, and anxiety relief.

When returning home, repeat this routine of exercises, meditation, and breathing. In your home environment, allow yourself to do what you want to do, eat or cook your favorite food, put on comfortable clothes, dance, sing, acknowledge that you are the comfort and peace these human beings need. You can speak with the NB, who still has no words. You understand their messages, conveyed by the body under your care. You welcome the parents at this delicate moment. You will also discover ways to take better care of yourselves in these demanding days because you are free to seek the best paths in life dynamics. We are thankful for the work you do. Thankful for your resilience and the teachings you share with our NB and their families.
